# The Year in Cardiology 2015: Prevention

**DOI:** 10.1093/eurheartj/ehv721

**Published:** 2016-09

**Authors:** M. John Chapman, Stefan Blankenberg, Ulf Landmesser

**Affiliations:** 1 National Institute for Health and Medical Research (INSERM), Pesquisa em Dislipidemia e Aterosclerose, Pitié-Salpêtrière University Hospital, Paris FR-75651, France; 2 University of Pierre and Marie Curie, Paris, França; 3 Clinic for Cardiology, University Heart Center Hamburg, German Center for Cardiovascular Research (DZHK), Hamburgo, Alemanha; 4 Departamento de Cardiologia, Charité Universitätsmedizin Berlin (CBF), Berlim, Alemanha; 5 German Center for Cardiovascular Research (DZHK), Berlin Institute of Health (BIH), Berlim, Alemanha

**Keywords:** Cardiovascular Diseases / prevention & control, Epidemiology, Coronary Diseases / therapy, Morbidity, Mortality

## Preamble

Improved prevention of cardiovascular disease (CVD) is of critical importance, as coronary heart disease (CHD) still represents the most common cause of death worldwide, engendering inestimable socioeconomic cost. The year 2015 has witnessed dramatic progress in CVD prevention on several fronts. Notably, this includes (i) event reduction in high-risk patients in general practice following introduction of a comprehensive strategy to attenuate modifiable risk factors, including lifestyle and dietary habits; (ii) the study of hybrid imaging to detect subclinical atherosclerosis, with potential improvement in risk prediction/management; (iii) the clinical demonstration, that culprit plaque rupture was observed in only 50-77% of patients with acute coronary syndromes; (iv) the emergence of 'omics' technologies to identify new causal biofactors; (v) the validation in clinical trials of the efficacy of monoclonal antibodies targeted to proprotein convertase subtilisin/kexin type 9 (PCSK9) in markedly reducing levels of low-density lipoprotein cholesterol (LDL-C) across a spectrum of patients at high risk of premature CVD, with preliminary findings strongly suggestive of reduction in cardiovascular events; (vi) significant reduction of cardiovascular and all-cause mortality in diabetic patients in the EMPA-REG OUTCOME trial with the anti-hyperglycaemic agent, empagliflozin, a selective sodium-glucose co-transporter-2 (SGLAT-2) inhibitor; (vii) new pharmacotherapeutic strategies for superior control of hypertension emanating from the PATHWAY-2 and PATHWAY-3 clinical trials involving spironoloactone add-on therapy in resistant hypertension, and amiloride plus hydrochlorothiazide in hypertensive patients requiring a diuretic, respectively; and finally (viii) a reduced mortality associated with a lower blood pressure target of 120 mmHg in patients at high cardiovascular risk in the SPRINT trial. Considered together, such progress augurs well for the future control of dyslipidaemia, hyperglycaemia, and hypertension, and with it, progressive reduction in atherosclerotic vascular disease and associated cardiovascular events in high-risk patients.

## Introduction

The prevention of CVDs represents an enormous challenge to health professionals on a global scale. Indeed, on the basis of the 2015 World Health Organization database for the European region, and calculating age-standardized mortality rates with the new European Standard population, CVD remains the most common cause of death among Europeans, accounting for 40% in males and 49% in females, and equating to > 4 million deaths per year.^1^ While mortality from CHD and stroke have decreased overall across Europe over the past decade, CHD continues to represent the single most common cause of death.^1^ Importantly, morbidity data reveal that population-based rates of hospitalization for both CVD and stroke have increased; considered together with ever increasing rates of cardiovascular interventions, greater use of medications, and expanding needs for rehabilitation for disabilities, these overwhelming socioeconomic costs present a major burden to healthcare systems across Europe.^1^

How can we address this insurmountable challenge? Clearly lifestyle and diet represent our first line of action as currently recommended in recent guidelines,^2,3^ and early identification and management of modifiable risk factors is paramount. Indeed, Avanzini *et al.*^4^ have recently demonstrated that application of a comprehensive personalized preventive strategy in > 12 000 high-risk subjects in general practice, but with suboptimal baseline risk factor control, led to gradual and significant improvement in global cardiovascular risk profile over a 5-year period. Thus, improvement in risk factor profile in the first year (including physical inactivity, hypertension, hypercholesterolaemia, diabetes, and an unhealthy diet) was independently and significantly associated with lower rates of cardiovascular events in subsequent years. These findings are entirely consistent with new observations from the EPIC-Norfolk prospective population study, in which even small improvement in modifiable risk factors led to substantial reduction in cardiovascular events.^5^ These important findings indicate not only that an integrated approach to modifiable risk factor control is feasible, but equally that it is achievable in general practice. Finally, imaging technologies for detection of subclinical atherosclerosis may be invaluable in adding incremental value to strategies for diagnosis, risk stratification, and early initiation of prevention (see below).

The year 2015 is - and continues to be - a vintage one for seminal progress in our knowledge of the pathophysiology underlying acute coronary syndromes (ACSs), and of the epidemiology, diagnosis, and prognosis of CVD, thereby reflecting concerted efforts in our quest to prevent the global scourge of atherosclerotic vascular disease and its thrombotic complications. Such advances have been paralleled by the successful and rapid development of highly efficacious, innovative therapeutics to markedly lower circulating levels of LDL-C. Indeed, in the landmark INTERHEART study of risk factors for the first myocardial infarction across 52 countries worldwide, atherogenic cholesterol transported as LDL predominated, accounting for the majority of population-attributable risk.^6^ In this context, it is especially relevant that recent genetic findings, involving Mendelian randomization strategies which integrate lifelong and therefore cumulative risk exposure, have consolidated the evidence base for a causal role of LDL in the pathophysiology of atherosclerosis and CVD^7-9^ (*Table* 1). Moreover, the IMPROVE-IT trial^10^ has now demonstrated that a mechanism of LDL lowering distinct from that of statins translates into clinical benefit. Ezetimibe-mediated inhibition of intestinal cholesterol absorption yielded incremental lowering of LDL-C on a background of statin treatment in this trial (involving 18 144 patients hospitalized for an ACS over 7 years) and translated into moderate improvement in cardiovascular outcomes, i.e. a 7.2% lower rate of major vascular events. Baseline levels of LDL-C were low (1.8 mmol/L or 70 mg/dL), with a 24% further reduction when ezetimibe was added to simvastatin; that cardiovascular benefit is proportional to the degree of LDL-C reduction is of critical relevance in this context.^11^ Cardiovascular mortality was not modified, a finding which may result from several factors, and particularly the need for post-trial, long-term follow-up data on clinical benefit. Indeed, it is increasingly evident that such follow-up reveals legacy benefits of LDL lowering beyond the active intervention period in randomized, placebo-controlled statin trials, typically featuring decrease in cardiovascular death rates.^12^ Clearly then, a new paradigm is appearing in which LDL lowering therapies may alter the pathophysiological course of atherosclerotic vascular disease and its thrombotic complications, potentially by inducing lesion stabilization, or lesion regression, or both.

**Table 1:** Evidence that LDL is causal in the pathophysiology of atherosclerotic vascular disease and cardiovascular events

• Epidemiology of risk factors for myocardial infarction, INTERHEART
• Familial hypercholesterolaemia
• RCTs with statins and ezetimibe (intestinal cholesterol absorption inhibition)
• Molecular genetics
– Mendelian randomization studies
– PCSK9 loss-of-function mutations and variants
– PCSK9 gain-of-function mutations
• Arterial lipoprotein retention and direct implication of LDL in plaque lipid accumulation
• Statin-mediated reduction in circulating LDL-C levels with concomitant decrease in plaque lipid and increase in extracellular matrix content, favouring plaque stabilization
• Plaque regression (reduction in atheroma volume) by statins

RCTs: randomized controlled trials; LDL: low-density lipoprotein; LDL-C: LDL cholesterol.

In this condensed distillate of advances in prevention of CVD over the past year, three key areas stand out. First, the evolution from emphasis on the ruptured, vulnerable coronary plaque to coronary plaque erosion in the context of ACS, with immediate relevance to approaches searching for 'vulnerable' plaques.^13^ Second, the appearance of advanced molecular methodologies for identification of biomarkers with potential for high predictive value.^14^ Third, the advanced development, based on the molecular genetics of familial traits for cholesterol dysmetabolism associated with premature atherosclerosis, of monoclonal antibodies targeted to PCSK9 for marked reduction in LDL-C levels.^15^ Importantly, progress in all three areas holds great promise to positively impact the care pathway for patients at high risk of CVD.

### Plaque imaging and cardiovascular risk prediction

A recent hybrid imaging study to evaluate the systemic extent of atherosclerotic disease in the carotid, abdominal aortic, iliofemoral, and coronary arteries in a middle-aged population (the PESA Study, Progression of Early Subclinical Atherosclerosis) revealed subclinical atherosclerosis in 63% of participants (71% men, 48% women), who ranged from low to high risk.^16^ With a similar approach, the BioImage Study (A Clinical Study of Burden of Atherosclerotic Disease in an At-Risk Population) evaluated the predictive value of carotid plaque burden (as examined by 3D ultrasound) and coronary artery calcification for cardiovascular risk assessment in a population of ~6000 asymptomatic adults who underwent multimodality vascular imaging of both coronary and carotid arteries. Both imaging methods suggested that higher detected plaque burden was associated with adverse cardiovascular events; furthermore, both imaging methods improved cardiovascular risk prediction to a similar degree.^17^

### Novel insights into coronary plaque pathobiology and mechanisms leading to progression towards acute coronary syndromes

Over recent years, coronary atherosclerotic plaque rupture and subsequent thrombus formation have been widely considered as the mechanism causing ACS. Subsequently, imaging studies have aimed to reveal the 'vulnerable plaque'. High-resolution intracoronary imaging studies using optical coherence tomography (OCT) have now revealed that a significant proportion of ACS events are caused by coronary plaque erosion (on an intact fibrous cap) and subsequent intracoronary thrombus formation, in addition to those 'classically' resulting from coronary plaque rupture of vulnerable thin-cap fibro-atheroma rich in lipid.^14^ Indeed, Libby and Pasterkamp^13^ have highlighted this consideration in an editorial entitled 'The requiem of the vulnerable plaque', in which they discuss different plaque pathobiologies leading to ACS. Moreover, Niccoli et al.^18^ reported that ACS caused by coronary plaque erosion may have a better prognosis as compared with those due to coronary plaque rupture, as such events appear to result from late thrombi suggestive of less intense thrombotic stimuli, thereby allowing time for thrombus dissolution caused by spontaneous fibrinolysis. Finally, a recent meta-analysis of OCT studies suggested that the mean prevalence of culprit plaque rupture and thin-cap fibro-atheroma was almost 50% across different clinical subsets of patients; importantly, such events were most prominent in ST-elevation myocardial infarction (70-77%).^19^

### Innovative methodologies for novel biomarker identification to assess cardiovascular risk

Although current risk models allow for increasingly precise risk equations in the general population, predicting life-threatening cardiovascular events at the level of the individual remains a challenge. More precise risk stratification, ideally based on causal factors, and personalization both of risk factor assessment and management are increasingly needed. A number of strategies have been employed to search for novel biomarkers of CVD. Unbiased technologies, including genomics, proteomics, and metabolomics, all utilize a 'big data' approach for novel biomarker discovery, but to date these technologies have failed to deliver on their initial promise, yielding no new clinically useful biomarkers in cardiac care. A genetic risk score has been analysed recently in clinical cohorts and data from randomized clinical statin trials and may identify individuals at increased risk for both incident and recurrent CHD events. People with the highest burden of this genetic risk derived the largest relative and absolute clinical benefit from statin therapy.^20^

An alternative strategy is to focus on known proteins reflecting mediating pathways to ensure a higher probability of association with CVD, an approach that can now be implemented on a massive scale using new multiplex immunoassay techniques that allow conservation of sample volume. This approach yielded promising results as recently tested in individuals with dysglycaemia.^21^ Further, non-coding RNAs including microRNAs are considered a potential biomarker, which might support diagnosis and prognosis in different cardiovascular conditions.^22^ Irrespective of big data approaches, single plasma biomarker assessment might be attractive to improve risk prediction models. Sensitive techniques to assess low concentrations of troponin I might open avenues to improve risk prediction in the general population by use of a cardiac-specific biomarker.^22,23^ Indeed, in the Bypass Angioplasty Revascularisation Investigation in Type 2 Diabetes trial, cardiac troponin T concentration measured with a high sensitivity assay was an independent predictor of death from cardiovascular causes, myocardial infarction, or stroke in patients who had both type 2 diabetes and stable ischaemic heart disease.^24^ Nevertheless, development of new strategies to identify causal biofactors is warranted in biological fluids, circulating cells, and tissues, and it is in this framework that emerging 'omics' technologies - metabolomics, lipidomics, proteomics, transcriptomics, and miRNAomics - augur well.^24^

### Prevention of atherosclerotic vascular disease and cardiovascular events in dyslipidaemia

#### Statin intolerance

**Figure 1:**
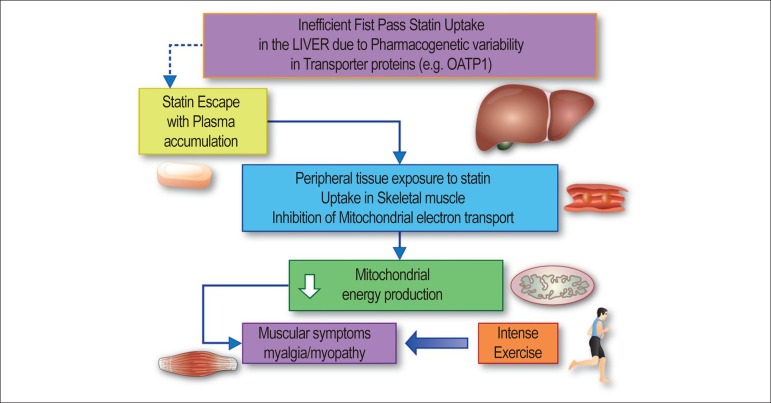
Statin-associated muscle symptoms predominate as adverse effects among dyslipidaemic subjects who discontinue statin treatment. Available evidence suggests that the pathophysiological basis for statin-associated muscle symptoms arises from inefficient uptake of statins by the liver, i.e. ‘statin escape’, frequently as a result of genetically determined variation in the structure of organic anion transporter proteins, such as organic anion transporting polypeptide 1 encoded by the SLCO1B1 gene. Thus, variant forms of the protein may exhibit low binding affinity for the statin. Under these conditions, first-pass hepatic uptake of the statin is incomplete, leading to elevated levels of statin in the circulation with prolonged residence time. At high statin doses, accumulation of statins in plasma correlates with a poor low-density lipoprotein cholesterol lowering response and a distinct trend to increased frequency of statin-associated muscle symptoms and myopathy.^25^ As a consequence, peripheral tissues such as skeletal muscle are exposed to high statin concentrations with the potential for enhanced uptake; several mechanisms appear to contribute to statin-induced reduction in ATP production and mitochondrial function in muscle cells.^25^ High demand for energy production in muscle, as occurs in intense exercise, may potentiate statin-associated muscle symptoms.

As recommended in current European guidelines, statins constitute first-line therapy in standard care for dyslipidaemic patients at high and very high cardiovascular risk in primary and secondary prevention.^2,3^ While the Cholesterol Treatment Trialists' meta-analyses of randomized controlled trials involving statins strongly substantiate their clinical efficacy,^11^ nonetheless, the profile of statin-associated adverse effects has been progressively clarified to reveal not only that statin-associated muscle symptoms (SAMSs) predominate in observational studies, registries, and clinical practice (range of prevalence 7-29%), but also that they are the primary cause of statin discontinuation.^25^ To this end, the European Atherosclerosis Society (EAS) Consensus Panel recently issued a statement providing clinical guidance in the form of a flow-chart for management of patients with SAMS, and recognized the central role of attenuated mitochondrial energy production in skeletal muscle in its pathophysiology; it is noteworthy that inefficient first-pass statin uptake into the liver may critically underlie SAMS (Figure 1).^25^ It is equally relevant that SAMSs are a central feature of 'statin intolerance', which also includes adverse events at the level of the liver, kidney, peripheral tissues, and potentially the central nervous system, but whose frequency is markedly less than that of SAMS.^25^

This Figure has been reprinted by permission of Oxford University Press on behalf of the European Society of Cardiology.

#### Inter-individual variability in response to statin therapy

Inter-individual variability in response to statin treatment has received little attention until late, when a pharmacogenetic meta-analysis of genome-wide association studies from randomized controlled trials and observational studies was reported, identifying the implication of two new genetic loci,*SORT1/CELSR2/PSRC1* and *SLCO1B1*, in addition to those of *APOE* and *LPA*, in variation in LDL-C response.^26^ These findings take on added significance when it is considered that a substantial proportion of patients with incident CHD are hypo-responders to statin therapy, show minimal LDL-C reductions, and most importantly, greater atheroma progression as compared with responders.^27^ Under such circumstances, follow-up monitoring of LDL-C levels after initiation of statin becomes primordial to ensure goal attainment.

#### Familial hypercholesterolaemia

Alarmingly, the proportion of patients with familial hypercholesterolaemia (FH) at LDL-C goal on statin treatment has been reported to be as low as 20% in the seminal Dutch experience; such patients are characterized by accelerated and premature atherosclerotic vascular disease and CHD.^28,29^ Several reasons may underlie this situation, some of which arise from the markedly elevated LDL-C levels frequently encountered at baseline in such patients. A maximally tolerated dose of an intensive statin is therefore the order of the day in FH, potentially in combination with ezetimibe, a synergistic association.^7,28-30^ Despite currently available therapies, however, FH in both its homozygous and heterozygous forms is widely underdiagnosed and undertreated, as emphasized by the EAS FH Consensus Panel.^28,29^ Indeed, the recent revelation from population genetic studies that FH is the most commonly inherited metabolic condition, with a population frequency approaching 1:200 persons, has warranted a call to action, with widespread creation of patient registries and FH patient advocacy groups.^28,31^ The under-diagnosis of FH is especially critical in children and adolescents, as emphasized recently by Wiegman *et al.*^31^ The evidence base in FH children treated with statins indicates not only that intervention with lipid lowering therapy may be safely initiated as early as 8 years of age, but also that when treated early in childhood, children born to FH families can anticipate normal life expectancy.^31^

#### The need for therapeutic innovation: PCSK9 inhibition

From the above, it is evident that innovative lipid lowering therapies have been-and remain-urgently needed, always on a background of statin treatment whenever possible, to fully translate the exceptional evidence base for reduction in cardiovascular events concomitant with LDL-C lowering into reality for many dyslipidaemic patients at high risk. Such patients include those with FH, those in secondary prevention, and those who are statin intolerant; additional patient populations may include individuals with diabetes, chronic kidney disease (CKD), and non-FH hypercholesterolaemia.^15^ It is in this context that the recent approval in the USA and Europe of two humanized monoclonal antibodies to PCSK9, alirocumab and evolocumab, is especially pertinent; the development of a third, bococizumab, which is partially humanized, is ongoing;^32^ all are well tolerated with a satisfactory safety profile.^15,33-35^ As exemplified by alirocumab, these antibodies act *in vivo* primarily by accelerating the fractional catabolic rate of LDL.^36^ An alternative approach to reduction of plasma PCSK9 concentrations involves direct inhibition of its hepatic production. A novel RNA interference drug, ALN-PCSsc (given as a subcutaneous formulation), has demonstrated the feasibility of this modality in phase 1 studies, resulting in a dose-dependent reduction in circulating PCSK9 levels of up to ≈80%, and a mean reduction in LDL-C of 40% for periods of 1 month or more, with favourable safety and tolerability.^37^

#### Monoclonal antibodies to PCSK9

The decade required for the development of monoclonal antibodies to inhibit PCSK9 has been driven by novel genetic and mechanistic insights into the role of this protein in the regulation of the availability of surface LDL receptors primarily in the liver, its relation to the regulation of circulating LDL-C levels, and ultimately to cardiovascular morbi-mortality.^38^ Quasi-complete removal of plasma PCSK9 by antibody binding results in highly efficacious lowering of LDL-C in the range of 40-70% as a function of dose across dyslipidaemic patient phenotypes in monotherapy or on a statin background, with uptake of LDL-antibody complexes by cells of the reticuloendothelial system; the duration of antibody action is dose-dependent for both alirocumab and evolocumab, whose (single dose) pharmacokinetics and pharmacodynamics resemble each other.^15,33,38^ Moreover, anti-PCSK9-mediated LDL lowering is additive to that of statins and ezetimibe.^15,33,38^ Importantly, the efficacy of these antibodies is independent of the specific class of the mutation of the LDL receptor (receptor negative, defective, unclassified, or no mutation detected) in heterozygous FH;^39^ this effect attests to the fact that PCSK9 action *in vivo* typically leads to the premature degradation of a major proportion of LDL receptors, a pathway largely neutralized by PCSK9 antibody treatment.^15^

In the 'Year in Cardiology 2014', De Backer *et al.*^40^ comprehensively reviewed extensive data from the phase III randomized controlled trials with alirocumab and evolocumab; clinical trial updates for 2015 are currently available in recent reviews.^15,38^ Of late, the ODYSSEY FH I and FH II (heterozygous FH) trials included the option to increase the antibody dose to 150 mg every 2 weeks when LDL-C goal was not attained on the starting dose (75 mg every 2 weeks). In this way, some 59-68% of patients achieved an LDL-C goal of < 1.8 mmol/L (70 mg/dL).^41^ Discontinuation due to treatment-emergent adverse events occurred in 3.4% of antibody-treated patients vs. 6.1% on placebo, while injection site reactions were reported for 12.4% in FH I and 11.4% in FH II ( vs. 11.0 and 7.4%, respectively for placebo), thereby attesting to satisfactory tolerability. Importantly and overall, these findings are consistent with those reported in FH heterozygotes upon treatment with evolocumab in the RUTHERFORD-2 trial, albeit involving a distinct dosing regimen from that above for alirocumab;^39^ furthermore, additional novel trial data have recently been reported in FH homozygotes in the TAUSSIG and TESLA trials (comprehensively reviewed by Chapman et al.^15^).

#### Safety of PCSK9 inhibition: vitamin E, gonadal hormones, cognitive function, very low LDL-C, and anti-drug binding or neutralizing antibodies

As lipophilic vitamin transport and steroidogenesis are intimately linked to LDL-C metabolism, it was critical to provide safety data for the potential impact of these innovative therapeutics on vitamin E and steroid hormone levels.^42^ Thus, in the 52 week, double-blind randomized placebo-controlled DESCARTES study, evolocumab, on a background of statin, did not affect gonadal hormone levels up to 52 weeks of treatment, while changes in vitamin E paralleled those in lipoproteins; erythrocyte vitamin E levels were unchanged.^42^ Equally, adrenocorticotrophic hormone (ACTH) levels and the cortisol/ACTH ratio did not change, even when LDL-C levels were very low (< 0.88 mol/L or 15 mg/dL).

Given that long-term statin therapy is associated with new onset diabetes, particularly in individuals presenting with features of prediabetes and the metabolic syndrome,^43^ it is imperative to exclude potential effects of PCSK9 inhibition on glucose homeostasis. Recent findings in the OSLER trial over a period of 52 weeks, involving subjects with impaired fasting glucose, metabolic syndrome and type 2 diabetes, demonstrate convincingly that PCSK9 inhibition (as evolocumab) was without effect on fasting plasma glucose and glycated haemoglobin (HbA1c) levels.^44^ Recent data with alirocumab equally indicate the lack of any adverse signal on glycaemic control.^45,46^

Practitioners frequently express two lingering concerns with respect to marked lowering of circulating LDL-C concentrations: first, low LDL-C levels may raise a range of safety issues; and second, prompted by concerns of the US Food and Drug Administration, low LDL-C on statin treatment may lead to deterioration of cognitive function. Importantly, patients who achieved very low LDL-C levels on statins displayed lower risk for major cardiovascular events.^47^ Furthermore, recent data from the OSLER trial have documented the absence of any safety signal as a function of on-treatment LDL-C levels down to 0.65 mmol/L (25 mg/dL).^38^ Similarly, ODYSSEY LONG TERM showed no increase in the incidence of AEs in patients attaining very low LDL-C levels (< 0.65 mmol/L or 25 mg/dL).^48^ Moreover, no significant signal concerning cognitive function has been detected to date in either the ODYSSEY or PROFICIO clinical trials programme.^34,35^ In addition, new findings from a Mendelian randomization study do not support a causal link between low LDL-C (< 1.5 mmol/L) and dementia, Parkinson's disease, or epilepsy.^49^ Notwithstanding these findings, the EBBINGHAUS trial, a substudy of the FOURIER outcomes trial, will examine the effect of evolocumab-induced low LDL-C levels on cognitive function using objective assessments.^50^ Finally, composite findings to date in the ODYSSEY and PROFICIO clinical trials programmes have revealed a very low incidence of anti-drug binding or neutralizing antibodies, involving 0.1-7.3% (placebo-corrected) of patients; the presence of such antibodies is typically transient.^34,45,41^ Long-term follow-up data will be essential to evaluate this key question fully, as it may equally be relevant to instances when a contingency for patients to switch antibodies may arise.

A word of caution is in order when considering the nature of 'very low LDL-C levels'. Typically, such levels are calculated on the basis of the Friedewald equation, and therefore include the cholesterol content of lipoprotein(a) [Lp(a)], thereby overestimating true LDL-C. In subjects with elevated Lp(a) levels and 'very low LDL', however, LDL may be effectively absent from plasma, and thus the readout potentially corresponds to Lp(a) cholesterol; the clinical implications of this concept are indeterminate.^51^ Under these conditions, ultracentrifugal isolation of LDL provides an accurate readout.

#### Cardiovascular outcomes trials

**Figure 2:**
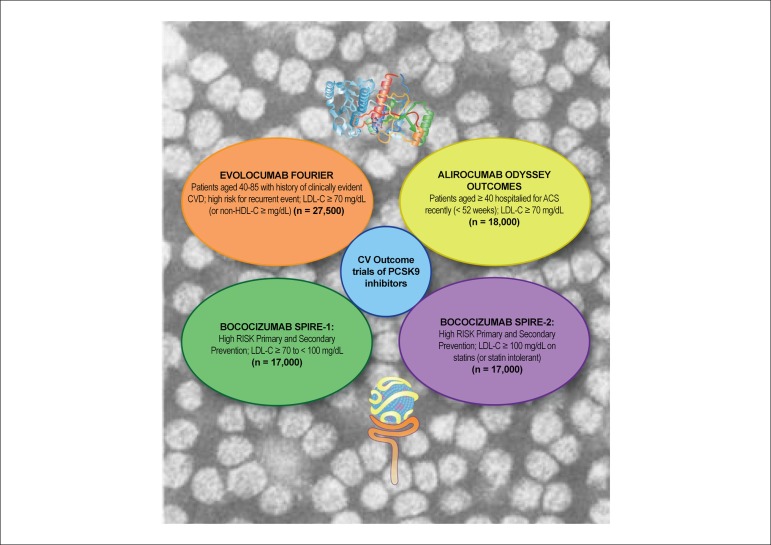
A schematic summary of the ongoing cardiovascular outcome trials for the three monoclonal antibodies to proprotein convertase subtilisin/kexin type 9, on a background of human LDL particles visualized by negative stain electron microscopy (copyright M.J.C.). The upper section of the figure shows a 2D image of the PCSK9 protein, while the lower section shows an image of an LDL particle bound to the biding domain of the LDL receptor. Overall, some 70 000 dyslipidaemic patients at high risk will be included in these multicentre, international trials. The primary endpoints in these trials, which are expected to report over the period of 2016–17 are as follows: FOURIER: cardiovascular death, myocardial infarction, hospitalization for unstable angina, stroke, or coronary revascularization, whichever occurs first;^56^ ODYSSEY OUTCOMES: coronary heart disease death, any non-fatal myocardial infarction, fatal and non-fatal ischaemic stroke, unstable angina requiring hospitalization;^57^ SPIRE 1 and SPIRE-2: major cardiovascular event, a composite endpoint that includes cardiovascular death, non-fatal myocardial infarction, non-fatal stroke, and hospitalization for unstable angina needing urgent revascularization.^54,55^ ACS: acute coronary syndrome; CV: cardiovascular; CVD: cardiovascular disease; HDL-C: high-density lipoprotein cholesterol; LDL-C: low-density lipoprotein cholesterol.

It is encouraging that exploratory analyses of ODYSSEY LONG TERM (alirocumab, n = 2341) and OSLER (evolocumab, n = 4465) indicate diminution in cardiovascular outcomes of 50-55% over treatment periods of up to 78 weeks.^44,48,52^ Moreover, a recent meta-analysis of 24 trials of PCSK9 antibody therapy, involving > 10 000 patients, highlighted a 55% reduction in all-cause mortality (p < 0.015), with similar decrements in cardiovascular mortality and myocardial infarction.^53^ Together with the SPIRE clinical trial programme for bococizumab,^54,55^ the FOURIER (patients with a history of CVD and at high risk of recurrent events)^56^ and ODYSSEY OUTCOMES (patients recently hospitalized for ACS)^57^ trials involve > 70 000 high-risk dyslipidaemic patients (Figure 2). While the findings are fully anticipated to confirm the preliminary observations discussed above, they will be essential elements in the evaluation of the long-term efficacy, tolerability, and cost-effectiveness of PCSK9 inhibition. We should not forget, however, that the trajectory of CVD over time is not limited to a single cardiovascular event, and that lowering LDL-C exerts cumulative, long-term arterial benefit, modifying the pathophysiological trajectory of atherosclerotic vascular disease.^12^ Therefore, critical appraisal of these agents should integrate their cumulative, long-term health benefits both for the individual and potentially for healthcare systems. In this light, we summarize future perspectives for PCSK9 inhibition in Table 2.

This Figure has been reprinted by permission of Oxford University Press on behalf of the European Society of Cardiology.

**Table 2:** PCSK9 inhibition: future perspectives

Cardiovascular outcomes from phase III trials
Impact on atherosclerotic vascular disease (Glagov imaging trial)
Impact of triglyceride-rich lipoproteins, remnant and lipoprotein(a) lowering, and HDL/apolipoprotein AI raising, on progression of disease and reduction in cardiovascular events
Long-term, real-life, safety data from post-marketing surveillance, including the safety of very low levels of LDL-C, and potential frequency of anti-drug binding or neutralizing antibodies
Evaluation of efficacy and safety in children and adolescents with heterozygous familial hypercholesterolaemia at high risk (the HAUSER-RCT trial)
Evaluation of efficacy in other patient populations at high risk, to include post-menopausal females, chronic kidney disease, type 1 and type 2 diabetics, peripheral arterial disease and autoimmune diseases
Use of PCSK9 antibody therapy to amplify and prolong LDL apheresis-mediated LDL-C lowering in severely affected familial hypercholesterolaemia patients, with potential to reduce frequency of apheresis treatment sessions
Evaluation of long-term cost-effectiveness as a function of long-term patient follow-up in individual healthcare systems

HDL: high-density lipoprotein; LDL: low-density lipoprotein; LDL-C: LDL cholesterol.

#### Beyond the LDL-C target: triglyceride-rich lipoproteins and lipoprotein(a)

In addition to LDL-C, PCSK9 inhibition, by virtue of its marked enhancement of LDL receptor number, may impact components of the atherogenic lipid profile beyond LDL-C, including triglyceride-rich lipoproteins and remnants (TGRL); such action may equally modulate levels of both high-density lipoprotein (HDL) and apolipoprotein (apo)AI via intravascular remodelling mechanisms. As exemplified by early results from OSLER, atherogenic TGRL levels are significantly reduced when PCSK9 is inhibited, while those of HDL/apoAI may increase;^4^ similar findings have been made across the ODYSSEY phase III studies.^34^ Further information on these actions as a function of baseline lipid profile will be of special interest, as we cannot exclude the possibility that they may enhance clinical benefit gained from LDL-C reduction alone.

The lack of therapeutic effect of statins on a potent atherothrombogenic lipid risk factor, Lp(a) has been perplexing, especially as abundant evidence now supports the contention that it is a causal, genetically determined and independent risk factor for premature CVD.^58,59^ Moreover, Mendelian randomization studies have documented a key role for Lp(a) in calcific aortic valve disease, an observation supported by new mechanistic insights intimately linked to its content of oxidized phospholipids.^60,61^ The finding then that PCSK9 inhibition reduces circulating Lp(a) levels by up to 35%,^62,63^ and that this effect may reside at least partially in the supra-physiological availability of LDL receptors for its catabolism, represents a major mechanistic advance.^64^ The ongoing cardiovascular outcomes studies for PCSK9 inhibitors may reveal whether Lp(a) reduction contributes to overall reduction in events. Ultimately, however, the answer to this question may require an outcomes trial involving antisense inhibition of hepatic apo(a) production in patients at high cardiovascular risk displaying elevated Lp(a) levels; such a scenario has entered the realm of possibility with the ongoing development of ISIS-APO(a) Rx, which can reduce Lp(a) concentrations by up to 80% dose-dependently.^65^

### Unmet clinical needs in dyslipidaemia: the therapeutic horizon

Clinical needs in moderate hypertriglyceridaemia are largely unmet to date, and are a central target on our therapeutic radar screen, especially the highly atherogenic mixed dyslipidaemia involving elevated levels of TGRL and subnormal HDL-C, a profile typical of insulin resistance.^66,67^ Molecular genetics has clearly identified the majority of such dyslipidaemic states as polygenic, upon which environmental influences are superimposed.^66,68^ Nonetheless, in the light of new genetic insights indicating that a loss-of-function mutation in apoCIII leads to concomitant fall in levels of TGRL and in cardiovascular risk, novel targeting of the apoCIII gene by antisense inhibition brings considerable optimism to this arena.^69^ Indeed, dose-dependent reductions attaining ≈80% in hypertriglyceridaemic patients (baseline triglycerides ~4.0-22.6 mmol/L or 350-2000 mg/dL) were found using a weekly injection protocol in phase II studies.^69^ No safety concerns were identified.

Patients with CKD are at high cardiovascular risk;^3^ preliminary findings suggest that PCSK9 inhibition is as efficacious in LDL-C lowering in those with moderate CKD as in those with mild or without CKD, with no evidence of safety issues.^70^

### Cardiovascular prevention in diabetes

After numerous cardiovascular outcome studies over the past years in patients with diabetes, suggesting no short- and medium-term risk reduction with anti-hyperglycaemic agents, the EMPA-REG OUTCOME trial reported a significant reduction of cardiovascular and all-cause mortality using a selective SGLAT-2 inhibitor, empagliflozin in patients with type 2 diabetes at high cardiovascular risk.^71^ These observations will have a significant impact on the future management of cardiovascular prevention in patients with type 2 diabetes.

### Novel insights into better control of hypertension

The PATHWAY-2 study has suggested that spironolactone is a particularly effective add-on drug for the treatment of resistant hypertension.^72^ The results of the PATHWAY-3 study support the first-line use of amiloride plus hydrochlorothiazide in hypertensive patients who need treatment with a diuretic.^73^ The DENERHTN study examined 106 patients with well-defined resistant hypertension and suggested that renal denervation plus an standardized stepped-care antihypertensive treatment (SSAHT) decreased ambulatory blood pressure more than the same SSAHT alone at 6 months,^74^ raising hope that renal denervation may lower blood pressure in well-selected patients.

Importantly, the SPRINT study^75^ demonstrated that among patients at high risk for cardiovascular events but without diabetes, targeting a systolic blood pressure of < 120 mmHg, as compared with < 140 mmHg, resulted in lower rates of fatal and non-fatal major cardiovascular events and death from any cause, although significantly higher rates of some adverse events were observed in the intensive-treatment group. This trial was larger than the previous ACCORD study, where a trend for a lower rate of cardiovascular events was observed with more intensive blood pressure lowering.

## Summary and conclusion

The year 2015 has seen dramatic progress in the control of dyslipidaemia, hyperglycaemia, and hypertension. These risk factors exert their nocivity throughout the course of the atherogenic process. Dyslipidaemia may, however, be unique as a target to attenuate progression of advanced plaques, and it is in this context that the marked efficacy of PCSK9 inhibition in lowering LDL-C to levels below the critical value of 1.8-2.1 mmol/L (70-80 mg/dL) required to stop progression in the majority of patients may present major therapeutic interest.^76,77^ Indeed, could rapid reduction of LDL-C to very low levels post cardiovascular event result in rapid lipid depletion and enhanced fibrous matrix content across diffuse plaques in the arterial tree, and with it, irreversible-or long-term-plaque stabilization with subsequent reduction in cardiovascular events? Could rapid attenuation of dyslipidaemia by PCSK9 inhibitors attenuate endothelial erosion on complex plaques, indirectly diminishing thrombotic complications? Such questions challenge cardiology, obliging us to determine the most efficacious pharmacotherapeutic strategies for CVD prevention. Finally, the first large cardiovascular outcome data of SGLAT-2 inhibition will have a major impact on the future treatment of diabetes, and in hypertension, the PATHWAY and SPRINT studies have provided valuable insights into optimization of treatment.
